# Large-Scale Whole Genome Sequencing Study Reveals Genetic Architecture and Key Variants for Breast Muscle Weight in Native Chickens

**DOI:** 10.3390/genes13010003

**Published:** 2021-12-21

**Authors:** Xiaodong Tan, Lu Liu, Xiaojing Liu, Huanxian Cui, Ranran Liu, Guiping Zhao, Jie Wen

**Affiliations:** 1 State Key Laboratory of Animal Nutrition, Institute of Animal Sciences, Chinese Academy of Agricultural Sciences, Beijing 100193, China; tanxiaodong08@163.com (X.T.); 18810610203@163.com (X.L.); cuihuanxian78@126.com (H.C.); liuranran112@126.com (R.L.); zhaoguiping@caas.cn (G.Z.); 2 College of Animal Science and Technology, College of Veterinary Medicine, Zhejiang A&F University, Hangzhou 311302, China; liulu@zafu.edu.cn

**Keywords:** chicken, missense SNP, breast muscle weight, genome-wide association study, *IGF2BP1*

## Abstract

Breast muscle weight (BrW) is one of the most important economic traits in chicken, and directional breeding for that results in both phenotypic and genetic changes. The Jingxing yellow chicken, including an original (without human-driven selection) line and a selected line (based on selection for increased intramuscular fat content), were used to dissect the genetic architecture and key variants associated with BrW. We detected 1069 high-impact single nucleotide polymorphisms (SNPs) with high conserved score and significant frequency difference between two lines. Based on the annotation result, the ECM-receptor interaction and fatty acid biosynthesis were enriched, and muscle-related genes, including *MYOD1*, were detected. By performing genome-wide association study for the BrW trait, we defined a major haplotype and two conserved SNPs that affected BrW. By integrated genomic and transcriptomic analysis, *IGF2BP1* was identified as the crucial gene associated with BrW. In conclusion, these results offer a new insight into chicken directional selection and provide target genetic markers by which to improve chicken BrW.

## 1. Introduction

For poultry, the weight and size of breast muscle are crucial to production efficiency and economic benefit by affecting carcass appearance, especially for chicken. Usually, the ratio of breast muscle weight (BrW) is as high as 20% or even 25% in fast-growing chickens (e.g., Cobb, Arbor Acres) [1,2], while it’s lower than 20% in slow-growing chickens [3,4]. Therefore, the identification of BrW associated genetic markers and causal genes is of particular importance to chicken breeding. The Jingxing yellow (JX) chicken is renowned for its delicious flavor, with meat rich in the major component of volatile organic compounds (hexanal and 1–octen–3–ol) [5]. These characteristics and advantages make JX chicken an important resource in breeding quality chicken. Unfortunately, this chicken has a slow growth rate and non-ideal muscle production (body weight <1500 g and eviscerated weight <1100 g at 90 d), which resulted in high aviculture costs. And the low meat production is prevalent in native chickens. Therefore, the foremost breeding goal for JX chicken is increasing meat production, especially BrW, with maintaining excellent meat quality and flavor.

Modern farm animals have been genetically adapted due to intensive human-driven selection, resulting in a genetically separate population that differs from the original population with regard to morphology, physiology, and behavior [6,7,8]. For the JX chicken, artificial selection for increased intramuscular fat (IMF) content in pectoral muscles has been undertaken since 2000 [9]. Significant phenotypic enhancement and a discrete genetic architecture were identified for this selected line in previous reports [9,10]. Given that IMF and BrW are related, BrW could be enhanced significantly along with the selection of high IMF in chicken [9,11]. Therefore, comparison of the original line and the selected line is an excellent means by which to dissect genetic architecture and explore the genes affecting BrW. Wang et al. revealed a new landscape of genomic changes associated with domestication by investigating the high-impact (missense) single nucleotide polymorphisms (SNPs), which were determined by low PROVEAN score [12]. Thus, it is conducive to identify the genes that respond to artificial selection by studying the missense SNPs in JX chicken.

Genome-wide association study (GWAS) is an efficient and precise method to inspect the genetic markers associated with complex traits (e.g., body weight, meat production, and fat deposition) in farm animals, especially for chicken due to the large-scale sequencing data [13,14]. For BrW trait, Liu et al. identified a significant genomic region (chr3: 61.82–68.57 Mb) affecting BrW in Beijing You chickens, with *NCO17*, *TPD52L1*, *FABP7*, *CJA1*, and *ASF1A* identified as candidate genes regulating BrW [15]. Kang et al. identified 43 genes that were correlated with BrW in transcriptional level for Tiannong partridge chickens [11]. Liu et al. reported chromosome 4 and 27 had BrW associated SNPs and genes (*LCORL* and *MAPT*), and both of these two genes were expressed at significantly higher levels in chickens with larger breast muscle [16]. These results suggest that candidate variants or genes differ among various subspecies, with no clear identification of the major functional genes related to BrW. However, the *IGF2BP1* gene has been studied widely in muscle development and lipid metabolism in animals [17,18]. Zhou et al. and Wang et al. demonstrated that *IGF2BP1* had a predominant role in body size as judged by GWAS and quantitative trait locus (QTL) mapping in duck and chicken, respectively [19,20]. In chicken advanced intercross line, an interval of chromosome 27 was recognized as a candidate genomic region (containing *IGF2BP1*, *GIP*, and other genes) that affected growth traits [13]. Taken together these results suggest a possible role for *IGF2BP1* in chicken breast muscle development.

Collectively, due to the changes of genetic architecture and BrW trait after selection of multi-generations [9,10], we analyzed the frequency and conservation of missense variants as a means by which to explore genomic alterations between original and selected chickens. GWAS and transcriptional analysis were then used to uncover genetic markers and candidate genes associated with BrW. In this manner genetic changes and pivotal markers associated with enhanced BrW, due to directional breeding, were identified.

## 2. Materials and Methods

### 2.1. Ethics Statement

All the experiments associated with chicken were conducted under the guidelines for experimental animals established by the Ministry of Science and Technology (Beijing, China). Ethical approval on animal survival was given by the animal welfare and ethics committee of the Institute of Animal Sciences (IAS, Beijing, China) and the Chinese Academy of Agricultural Sciences (CAAS, Beijing, China) with the following reference number: IAS2019-21.

### 2.2. Animals

Female JX chickens (total *n* = 520) were obtained from the Chinese Academy of Agricultural Sciences, Institute of Animal Sciences (Beijing, China). Of these, 264 JX chickens (the selected line) were used. This line had been propagated for 16 generations for enhanced breast intramuscular fat content [21]. As well, 256 JX chickens (the original line) were used. This line had not undergone any human-driven selection [9,10,21]. All chickens were fed the same basal diet formulated according to NRC (1994) and NY/T (33-2004), and raised in three-story step cages (one chicken per cage) with the recommended environmental conditions. Feed and water were provided ad libitum during the study.

### 2.3. Phenotypic Measurement

The chickens were weighed at 98 days of age after 12-h of fasting, euthanized, and then exsanguinated by severing the carotid artery. The whole breast muscle (major and minor breast muscle) was removed, weighed, and BrW was recorded (Supplement Table S1). Venous blood samples were also collected and stored at −20 °C for DNA extraction.

### 2.4. Genotyping, Imputation, and Quality Control

Genomic DNA was isolated from blood samples (*n* = 520) by the phenol-chloroform method. After quality control by gel electrophoresis, eligible DNA (presenting obvious strap and no degradation) was used to construct a pair-end library of 300–500 bp of average insert size. Each library was sequenced with a Novaseq 6000 sequencing platform (Illumina, San Diego, CA, USA) to acquire raw reads with an average depth of 10× coverage. The data can be accessed at CRA002643 and CRA002650 (https://bigd.big.ac.cn/gsa/) (accessed on 10 October 2021) [21]. Variant calling was implemented according to the standard bioinformatic pipeline [22,23]. Briefly, the Burrows Wheeler Aligner MEM algorithm [24] was used to align the clean reads to the reference genome GRCg6a (http://ftp.ensembl.org/pub/release-104/fasta/gallus_gallus/dna/Gallus_gallus.GRCg6a.dna_sm.toplevel.fa.gz) (accessed on 10 October 2021). The output was converted to bam files and sorted by coordinates based on Samtools software [25]. PCR duplication was deleted with the default parameter of Picard software. SNP calling was executed with the HaplotypeCaller function, and GVCF format variant files were obtained with the Genome Analysis Toolkit (GATK 3.6) [26]. The GVCF files of each individual were combined and joint variant calling was performed by GenotypeGVCFs function. Finally, the VariantFiltration function in GATK was used to acquire qualified SNPs with the following parameters: variant confidence score < 30.0, QualByDepth < 2.0, ReadPosRankSum < −8.0, total depth of coverage < 4.0, FisherStrand > 60.0.

The SNPs located in the autosome and the Z chromosome were reserved for next analysis. Quality control was implemented with the particular parameters (--mind 0.2, --geno 0.1, --maf 0.05) in PLINK 1.9 software [27]. After quality control, linkage disequilibrium phasing was conducted to impute the missing alleles with the default parameter in Beagle 5.0 [28]. Ultimately, a total of 9,722,764 SNPs and 515 birds were retained (three chickens were removed due to abnormal phenotypic record and two chickens were removed due to lower call rate).

### 2.5. SNP Annotation, Frequency, and Conservation Analysis

SnpEff (v.5.0) was used to annotate variants based on their location categorization [29]. Conservation analysis was evaluated by the PhastCons score using SnpSift software (v.5.0) [30], PhastCons information can be downloaded from the UCSC database (http://hgdownload.soe.ucsc.edu/goldenPath/galGal6/phastCons77way/galGal6.77way.phastCons/) (accessed on 10 October 2021). To compare the allele frequency difference between original and selected chickens, we calculated the frequency of missense variants in original and selected lines, respectively. And Fisher’s exact test was used to calculate the *p* values [31], and the Bonferroni method was used to determine the genome-wide threshold for missense variants.

### 2.6. Heritability Estimate for BrW

To estimate the heritability for BrW, we firstly calculated the kinship matrix by the method of VanRaden using the GCTA software [32,33]. And then, the variance component and heritability were estimated by the univariate animal model constructed by ASReml v4.1 in R environment [34]. The univariate animal model was defined as follows:

y=Xb+Za+e,

where *y* indicates the BrW phenotype, *b* represents the vector of batch effect, *a* represents the vector of random additive genetic effects, *e* represents the vector of random residual effect. *X* and *Z* are design matrices related to the corresponding fixed and random effects, respectively.

### 2.7. Genome-Wide Association Study

An univariate linear mixed model (LMM) was used to perform association analysis based on all SNPs for BrW in GEMMA (0.98.4) software [35]. Within LMM, genotype was presented as the fixed factor and the additive polygenic effect as the random effect for exploration of marker effects and significance. The centered kinship matrix was calculated based on all SNPs. Batch effect and strain effect were used to assess the influence on BrW via the general linear model in R. The two effects were both included in LMM as the fixed effect. The LMM emerged as follow:

y=Wα+xβ+μ+ϵ,

where *y* represents the vector of BrW record; W represents the matrix of covariates, including a column of 1s, as well as batch and strain effect; α is vector of the corresponding coefficient (including the intercept); β is the effect size of each marker; $\mu \sim MVN_{n}\left(0,\;\lambda \tau^{-1}K\right)$, μ represents the random polygenic effect, $MVN_{n}$ denotes the n-dimensional multivariate normal distribution, λ is the ratio of two variance components, $\tau^{-1}$ is the variance of the residual errors, K is the kinship matrix; $\epsilon \sim MVN_{n}\left(0,\tau^{-1}I_{n}\right)$, ϵ is the residual error, $I_{n}$ is the identity matrix. A Wald test was conducted to estimate the significance of markers related to BrW. A restricted maximum likelihood algorithm was used to evaluate λ and β.

To reduce the false positive probability, a Bonferroni correction was used in association analysis. A genome-wide and suggestive threshold were defined as 5.14 × 10^−9^ (0.05/9,722,406) and 1.03 × 10^−7^ (1/9,722,406), respectively. Genomic inflation factor (λ) was calculated to estimate bias and stratification. The proportion of variance explained by lead SNP was evaluated as the equation: $\frac{2pq\beta^{2}}{\sigma_{g}^{2}}$ [36], where p and q represent allele frequency for minor and major alleles respectively, β represents the allele effect size, $\sigma_{g}^{2}$ represents the genetic variance, which calculated by ASReml 4.1. Manhattan and quantile-quantile plots were visualized via the qqman package in the R environment [37].

### 2.8. Narrowing the Candidate Region and Gene Annotation

Pairwise r^2^ for the lead SNP was calculated in PLINK 1.9 [27]. A narrowed interval was defined with r^2^ > 0.8 and the 2-LOD drop-off method [38]. A significant SNP was phased to infer the haplotype that may harbor the causal mutation. GLM and least significance difference method of multiple comparisons were used to evaluate the haplotype effect on BrW. The PhastCons conserved score based on 77 vertebrates was used to detect the conserved SNP within the candidate region. The candidate genes were defined if located approximately 10 kb upstream and downstream of the narrowed region. Candidate region and genes were annotated by biomaRt [39].

### 2.9. Transcriptomic Analysis Based on Multiple Stages and D98

The transcriptome data were acquired with accession number CRA001334 (sampling from the generation 15 of JX chickens) [40] and CRA001908 (sampling from the same generation as GWAS) [41]. The raw reads were filtered with Trimmomatic 0.36 software (LEADING:3 TRAILING:3 SLIDINGWINDOW:4:15 MINLEN:50) [42], and acquired clean reads were mapped to reference genome GRCg6a (http://ftp.ensembl.org/pub/release-104/fasta/gallus_gallus/dna/Gallus_gallus.GRCg6a.dna_sm.toplevel.fa.gz) (accessed on 10 October 2021) using HISAT2 [43]. Then, we sorted and indexed the bam file, and extracted the high quality mapped reads for assembling and merging the gtf files using StringTie 2.1.6 [44]. Here, we only focused on the known transcript and skipped the assembling process of novel transcripts with -e parameter. Based on the merged gtf file, we assembled it secondly and calculated the raw count of each gene. The differentially expressed genes (DEGs) analysis between the same original and selected populations as GWAS was conducted by DESeq2 software in R environment [45], and DEGs were defined as fold change (FC) > 1.5 or FC < 0.67 and adjusted *p* value < 0.05. Stage-based expression level for candidate genes were presented as adjusted count value using the data from generation 15.

### 2.10. KEGG Pathway and GO Term Analysis

KEGG pathway enrichment analysis was performed by ClusterProfiler [46] to explore the function of genes, which were annotated by missense SNPs with frequency difference or high conservation score. Also, the KEGG and GO analysis were performed based on DEGs between original and selected population. A *p* value of 0.05 was regarded as the threshold for significant enrichment.

### 2.11. Statistical Analysis

GLM was used to estimate the significance of the effect of lead SNP on BrW. Pearson correlation analysis was used to evaluate the relationship between gene expression and BrW.

## 3. Results

### 3.1. Genomic Variants Annotation

**Figure 1:**
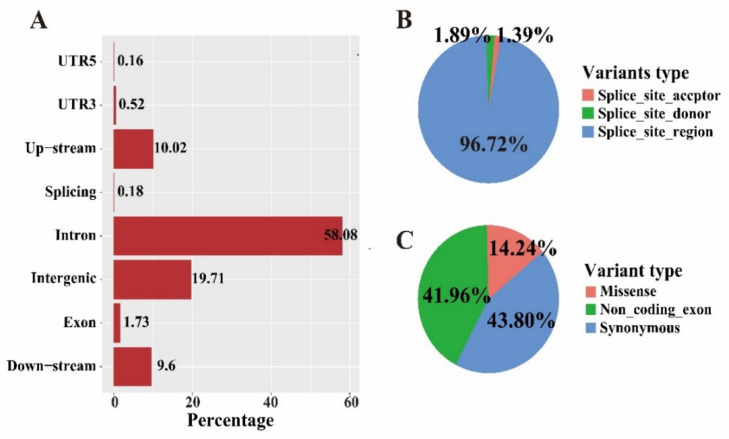
SNP characteristics of JX chicken. (**A**) Distribution of genetic variations within various function regions. (**B**,**C**) Ratio of different variants in splicing site and exon region, respectively.

Bioinformatics analysis using the described pipeline detected over 9.7 million high-quality bi-allelic SNPs in autosome and Z chromosome after filtration. The retained variants were distributed in the genome with an average density of 1 SNP every ~110 bases. Among those, about 58.08% of the SNPs were located in intron region, with only 1.73% of SNPs anchored in exons (Figure 1A). Approximately 10.0% were up-stream and 9.6% were down-stream of genes, respectively. For the SNPs around splicing site, an extremely low ratio of splicing site acceptor (1.39%) and donor (1.89%) variants was detected (Figure 1B). For the SNPs in exons, the synonymous (43.80%) and non-coding exon (41.96%) variants were predominant, while the missense variants only accounted for 14.24% (Figure 1C).

### 3.2. Allele Frequency Spectrum

**Figure 2:**
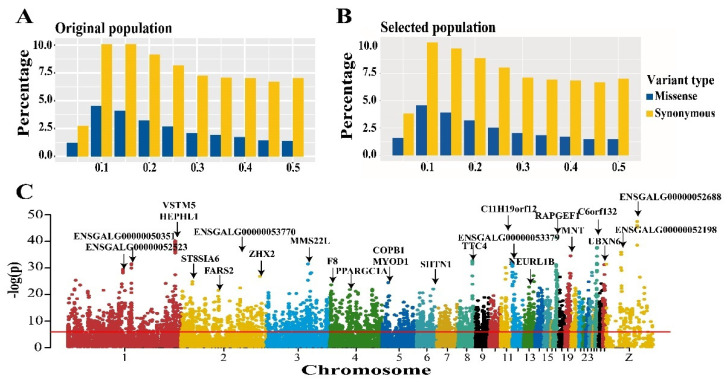
Analysis of allele spectrum and frequency difference of missense and synonymous SNPs. (**A**,**B**) Minor allele frequency spectrum of missense and synonymous SNPs in original and selected population. (**C**) Manhattan plot of frequency difference of missense variants between two lines, the ***y***-axis represents the *p* value in log scale, which calculated by Fisher’s exact test, the ***x***-axis represents the physical position of missense SNPs in each chromosome.

Comparison of the allele frequency profiles of original and selected populations revealed no obvious differences (Figure 2A,B). A decreasing percentage was observed with increasing minor allele frequency (>0.10) in both original and selected chickens. Considering the high impact of missense variants on protein function, the Fisher’s exact test was used to detect the significance of allele frequency difference between original and selected chickens, and the Bonferroni method was used to determine the genome-wide threshold (*p* < 1.54 × 10^−6^). A total of 5352 significant missense variants were detected, primarily in 1–4 chromosomes (over 40%, Figure 2C, Supplement Table S2). To evaluate the potential biological effect of these significant missense variants, we annotated them using biomaRt and performed KEGG enrichment analysis based on hypergeometric distribution for the acquired genes. A total of 2840 protein-coding genes were annotated based on significant missense variants (Supplement Table S3). Some of these were involved in multiple functional KEGG categories, including glycosaminoglycan degradation, ECM-receptor interaction, and fatty acid biosynthesis (Supplement Figure S1 and Supplement Table S4).

### 3.3. Conservation Score Analysis 

**Figure 3:**
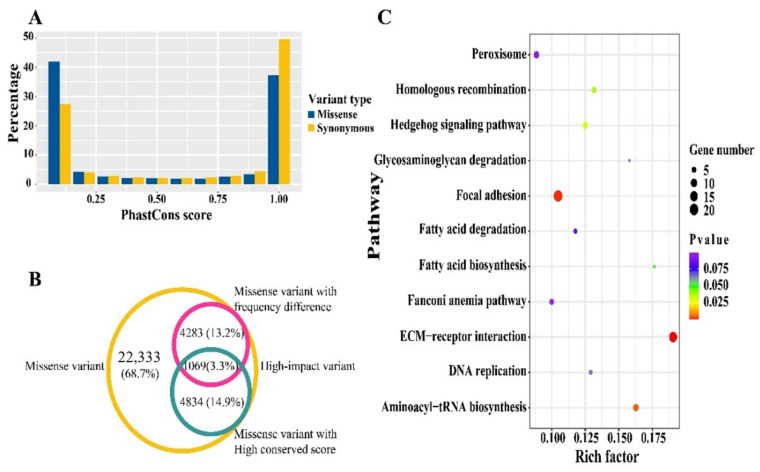
Conservation analysis, annotation and enrichment analysis for high-impact variants. (**A**) Ratio of variants with various conservation score. (**B**) Overlapping of different types of missense variants, the missense variants with frequency difference and high conserved score were defined as the high-impact SNPs. (**C**) KEGG pathways enriched by genes harboring high-impact SNPs.

Genomic functional sites usually tend to be evolutionary conserved. Therefore, we evaluated the conservation status for each synonymous and missense variant by basewise PhastCons conservation score using the snpSift component for 77 vertebrates. A similar bipolar pattern for distribution of PhastCons score was exhibited between synonymous and missense variants. Over 75% of the SNPs had conservation scores ranging from 0–0.1 or 0.9–1.0 (Figure 3A). To explore the latent high-impact variants, we focused on the conserved site with a PhastCons score greater than or equal to 0.98. A total of 1069 conserved variants were overlapped with previous missense sites of significantly different frequency between original and selected chickens (Figure 3B, Supplement Table S5). Of these, 912 protein-coding genes were annotated based on the overlapped variants (Supplement Table S6). Some of the acquired genes were included in ECM-receptor interaction, focal adhesion, and fatty acid biosynthesis pathways (Figure 3C, Supplement Table S7). It’s worth noting that partial genes involved in skeletal muscle growth and development were detected, including the marker gene of slow-twitch *MYH7* and crucial myogenic process transcription factor *MYOD1*, as well as some members of the MYO gene family (e.g., *MYO1D*, *MYO1H*, and *MYO18B*) (Supplement Table S6). 

### 3.4. GWAS for BrW

**Figure 4:**
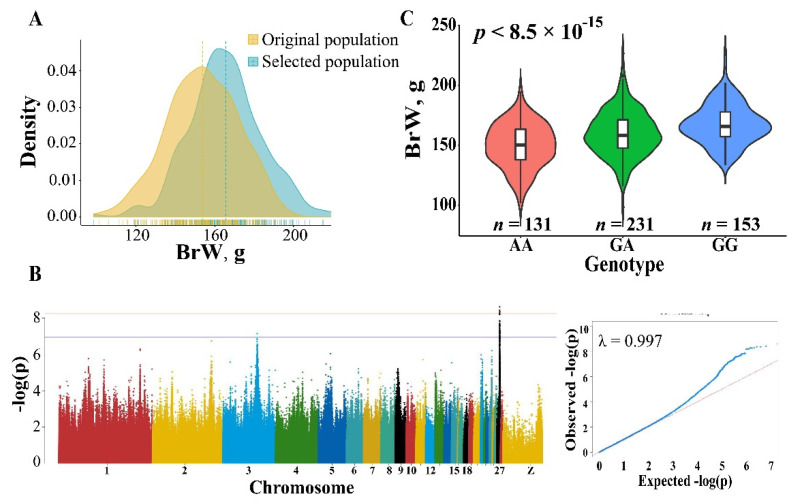
Phenotypic difference and GWAS result for BrW. (**A**) Comparison for BrW in original and selected population. (**B**) Manhattan and quantile-quantile plot for BrW. A significant region was identified in chromosome 27 (the black dots). The horizontal red and blue lines represent the genome-wide and suggestive significance, respectively. (**C**) The effect of the lead SNP chr27_6115361 on BrW, the *p* value was calculated using GLM.

Based on the results that the vital genes related to muscle development were high-impact variants, we compared BrW between original and selected population. As expected, we found that the heritability of BrW is relatively high (h^2^ = 0.35) and higher BrW was observed in the selected population (Figure 4A) with a nearly 8% difference found between the original and selected population. To find the candidate variants and genes affecting BrW, we performed GWAS for this trait using LMM. In the 6.08–6.33 Mb of chromosome 27, an extremely significant peak was detected (Figure 4B). Within this region, 63 significant SNPs (*p* < 1.03 × 10^−7^) were found. The proportion of variance in genotypes was explained by the lead SNP (chr27_6115361) at up to 18.93% (Supplement Table S8). The minor allele frequency of those significant sites was from 0.27 to 0.50 (Supplement Table S8). To maximize detection efficiency of candidate genes, we expanded the up- and down-stream candidate interval to 10 kb (Table 1). In this manner, 17 protein-coding genes and 2 miRNA were identified, including *IGF2BP1*, *GIP*, and the members of *HOXB* gene family (e.g., *HOXB2*, *HOXB3*, and *HOXB4*). In addition to the peak in chromosome 27, only one significant SNP greater than threshold was observed on chromosome 3. Located 10 kb up-stream of this site, lncRNA (*ENSGALG00000034564*) is the only annotated gene in this region by the current genome build (Table 1).

### 3.5. Narrowing the Candidate Region

**Figure 5:**
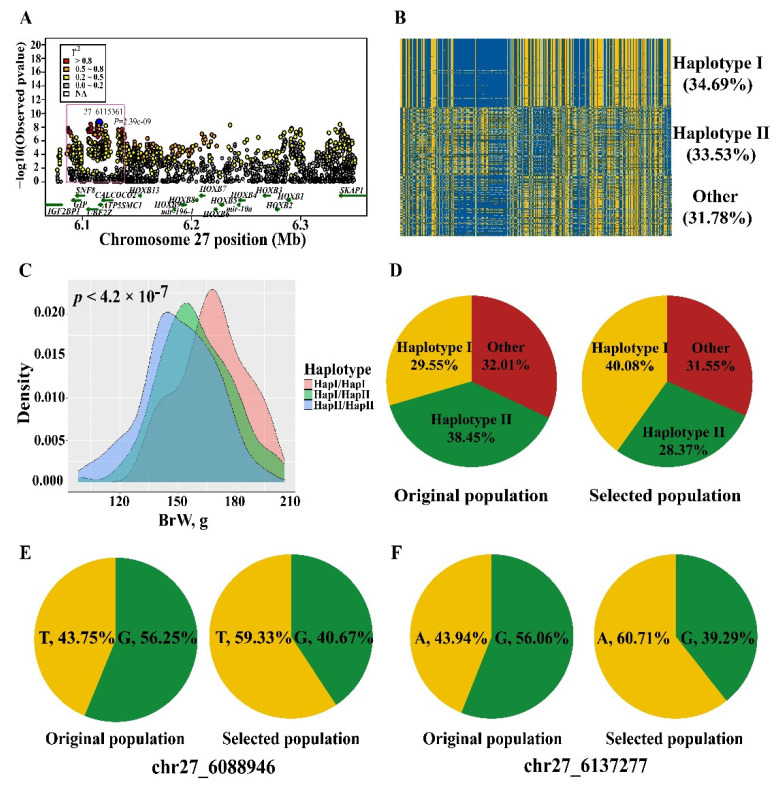
Haplotype association analysis and frequency difference in original and selected populations. (**A**) A refined genomic region was identified by r^2^ > 0.8 and 2-LOD drop-off method, namely the pink box. (**B**) Distribution of two major haplotypes (I and II) in JX chicken. (**C**) The effect of major haplotypes on BrW, the haplotype I has an increasing effect on BrW. (**D**) The frequency of two major haplotype (I and II) in original and selected population. (**E**,**F**) The frequency of two conserved sites (chr27_6088946 and chr27_6137277) in original and selected population.

To investigate the BrW regulatory interval, we narrowed the candidate region by defining r^2^ > 0.8 and the 2-LOD drop-off method. A 51-kb refined region, chr27: 6.08–6.14 Mb, was identified with a total of 31 candidate SNPs (Figure 5A, Supplement Table S8). To find causal mutations, we inferred the haplotypes formed by those 31 SNPs and found 2 major haplotypes (frequency > 30%) (Figure 5B, Supplement Table S9). The effect of the haplotypes was evaluated by GLM, with haplotype I associated with enhanced BrW compared to haplotype II and the heterozygous haplotype state (*p* < 4.2 × 10^−7^, Figure 5C). The frequency of the three genotypes (homozygote for haplotype I or II, and heterozygote) was assessed for original and selected chickens, with greater frequency for haplotype I (Figure 5D). The site conservation score was determined as described above. Only two SNPs (chr27_6088946, chr27_6137277) were highly conserved in vertebrates, with significant differences between the two lines (Figure 5E,F), indicating an important role in regulation of BrW. The refined region contained *IGF2BP1*, *GIP*, *SNF8*, *UBE2Z*, *ATP5MC1*, and *CAOCOCO2*, and chr27_6088946 was located in the up-stream of *IGF2BP1* and *GIP* (Figure 5A).

### 3.6. Identification of Candidate Genes by Transcriptome Analysis

**Figure 6:**
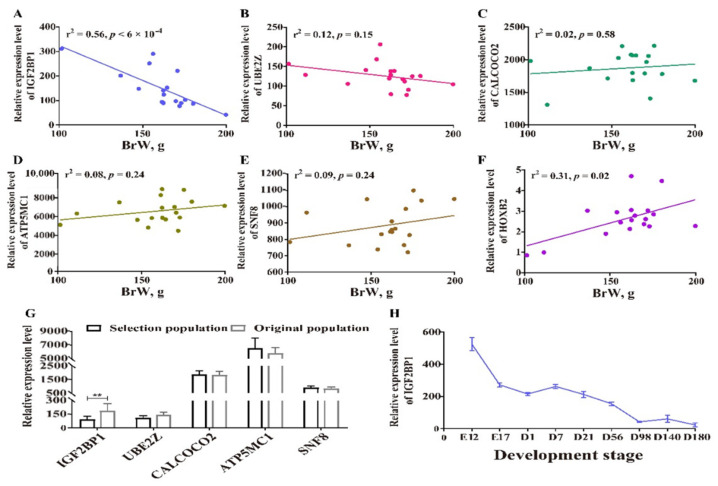
Transcriptomic analysis for candidate genes. (**A**–**E**) Correlation analysis between BrW and genes *IGF2BP1*, *UBE2Z*, *CALCOCO2*, *ATP5MC1*, and *SNF8*, respectively. (**F**) Comparison for BrW from the individuals performing the RNA-seq (*n* = 9 each population). (**G**) Differentially expressed genes analysis for *IGF2BP1*, *UBE2Z*, *CALCOCO2*, *ATP5MC1*, and *SNF8* between two lines, ** indicates *p* < 0.01. (**H**) Stage-based expression pattern of *IGF2BP1*, E indicates embryo stage, D indicates the age of after hatching.

To maximize mining of candidate genes, all the genes located within and outside the defined region chr27: 6.08–6.14 Mb were both included in the following transcriptional analysis. First, we correlated the expression level of those genes with BrW, and the only significant results were for *IGF2BP1* and *HOXB2* expression, which were in negative and positive relationship with BrW, respectively (Figure 6A–F, Supplementary Figure S2). To determine whether altered transcriptional levels were due to human-driven selection, we next compared the gene expression between original and selected chickens. The genes *IGF2BP1*, *HOXB4*, *HOXB5*, *HOXB6*, and *HOXB7* were differentially expressed between the two lines, whereas *SNF8*, *ATP5MC1*, *UBE2Z*, *CALCOCO2*, and other members in HOXB gene family were not differentially expressed (Figure 6F,G, Supplement Figure S3). Therefore, *IGF2BP1* could best be seen as plausible candidate gene, and so we examined dynamic expression pattern for *IGF2BP1* during different stages (Figure 6H). An obvious decreasing expression of *IGF2BP1* was found from E12 to D180, which was negatively related to the development pattern of breast muscle (Supplement Figure S4).

In addition, we defined 2494 DEGs (482 down-regulation and 2012 up-regulation) between two lines (Supplement Figure S5, Supplement Table S10). By enrichment analysis, muscle related pathways (e.g., tight junction, calcium signaling pathway) and GO terms (e.g., muscle cell differentiation, muscle structure development) were determined as significant (Supplement Figures S6 and S7), indicating that the genes and pathways potentially regulating BrW in the selected line were significantly differentiated from that in the original line in transcriptomic level.

## 4. Discussion

JX is a widely known domestic chicken breed due to its excellent meat quality. Regarding pectoral IMF content plays an essential role in meat quality, we constructed a 16th generation IMF selected line [9,10,21]. For indigenous chicken, meat production, especially BrW, is the cardinal breeding index. In this study, a significant increase in BrW (about 12 g) was found in the IMF selected population, which was consistent with a previous report that a mild correlation was found between chicken IMF and BrW [11]. Herein, we used this ideal chicken model to investigate genetic markers associated with BrW.

Long-term artificial selection of farm animals can produce biased genetic variants or genomic signatures that distinguished the selected animals from the original population, which may be caused by pleiotropy of genes under selection, hitch-hiking of unfavorable alleles with the alleles under selection [7,8]. Therefore, we assessed the selection effects on the genome before exploring the genetic markers associated with muscle development. A distinct pattern based on global genomic variants identified a clear divergence between the selected and original lines [10]. Furthermore, a parallel allele frequency pattern, based on synonymous and missense variants, was obtained that was consistent with observed patterns in humans and cattle [47,48]. Wang et al. defined high-impact SNPs as having a PROVEAN score < −2.5 for missense variants [12]. Herein, we focused on frequency changes between the two chicken lines and conservation status, to evaluate chicken phenotype high-impact variants rather than on PROVEAN score for missense SNPs. After strict quality control (*p* < 1.54 × 10^−6^, Fisher’s exact test for allele difference, PhastCon score > 0.98), 1069 missense SNPs and 912 protein-coding genes were found to influence chicken traits during intensive selection. This result indicates that only a small percentage of genomic sites responded to artificial selection or possible environmental effect [49]. Based on the above protein-coding genes, lipid metabolism related pathways, ECM-receptor interaction, and fatty acid biosynthesis, were enriched in the selected chicken line [41]. 

In a previous study, it has been demonstrated that the BrW was elevated after selection of IMF [9]. Here we found a divergent pattern for the genes related to muscle development in genomic and transcriptomic levels, including myosin complex genes (e.g., *MYO1D*, *MYO1H*, and *MYO19*), *MYH7*, *MYOD1*, *MYF5*, and other genes (Supplement Table S10). Therefore, we performed GWAS to explore genetic markers associated with BrW. To minimize false positives, Bonferroni correction method was used and identified a 260-kb region (chr27: 6.08–6.34 Mb). By annotation, *IGF2BP1*, *GIP*, *SNF8*, *UBE2Z*, *ATP5MC1*, *CALCOCO2*, *HOXB* gene family members, and two microRNAs were found in the region. Wang et al. have reported that *IGF2BP1* affected chicken body size (e.g., claw weight, shank length, and carcass weight), and a deletion mutation in the promoter region has been validated as a causal variant [20]. Similarly, *IGF2BP1* was shown to affect body size in ducks [19]. In this study, the candidate region was located in the promoter and up-stream region of *IGF2BP1*, which is consistent with the previous report [20]. Further, *GIP* is important to bone size and growth traits in chicken [13,50], but zero expression was detected in muscle. *HOXB5* was differentially expressed between two lines, but the correlation with BrW was relatively low. The genes *HOXB5*, *HOXB6*, and *HOXB7* were involved in the morphogenesis of chicken respiratory tract [51], *HOXB7* and *HOXB8* were also shown to be associated with beard trait in chicken [52], but few reports suggested a role for *HOXB* genes in breast muscle development. Although *HOXB2* was positively correlated with BrW, the expression level of which is relatively low in muscle tissue. In addition, we detected no expression of *HOXB1*, *HOXB9*, *HOXB13*, *SKAP1* in muscle tissue, as well as the lncRNA (*ENSGALG00000034564*). According to expression atlas, the microRNAs gga-mir-10a and gga-mir-196 were not expressed in skeletal muscle [53]. *SNF8*, *UBE2Z*, *CALCOCO2*, and *ATP5MC1* have been shown a core function in metabolic disease and cancer. Partial QTLs or SNPs have been associated with coronary artery disease (*SNF8* and *UBE2Z*) [54,55]. *CALCOCO2* is associated with autophagy-related genes, influencing tumorigenesis and progression of osteosarcoma [56]. Overexpression of *ATP5MC1* was confirmed to perturb glucose metabolism and inhibit the oncogenic K-Ras signaling [57]. But no studies have reported that these genes were involved in the growth process of chicken.

Current knowledge of these genes makes identification of causal variants and associated genes difficult. To narrow candidate genomic regions and ensure accuracy, we combined the linkage status of the lead SNP with the 2-LOD cutoff method for fine-signal mapping [38]. A 51-kb refined region and a major haplotype comprising 31 significant SNPs were identified, which enhanced BrW by 10%. Liu et al. reported significant regions for the percentage of breast muscle (BrP) in chromosome 1, 10, and Z of Cobb × Beijing You F2 population [16]. Liu et al. defined prominent SNPs associated with BrW and BrP in chromosome 3 of Beijing You chickens [15]. Pampouille et al. identified multiple genomic regions (e.g., chr4, 5, and 8) interrelated with breast phenotypes in high and low pH selection lines [58]. The range of identified loci associated with BrW in different chickens is relatively large due to low density of marker. In this study, we identified a narrowed genomic region affecting BrW based on a whole genome sequencing strategy, which provided a target by which to investigate the mechanisms associated with BrW in chickens.

In this region, the frequency of the major haplotype and two conserved SNPs were consistently changed toward directional selection. These results provide evidence for functional selection of an animal genome within a limited number of years [59]. However, a functional assay to verify the role of conserved SNPs is still required. To precisely map the candidate genes, we integrated transcriptome data to investigate the expression level of those genes from previous studies [40,41]. We found that only the mRNA level of *IGF2BP1* was negatively related to BrW. Wang et al. reported higher transcription level of *IGF2BP1* was detected in Ross compared with that in Gushi chickens [20]. This indicates that body size and muscle development are likely regulated in a positive manner by *IGF2BP1*. But actually, cellular assay demonstrated that reinforced proliferation of myocytes was observed with si*IGF2BP1* treatment in C2C12 myoblasts, and a decreasing trend was captured during the prenatal stage (E33~E95) of skeletal muscle development [60]. Those results are highly consistent with the results reported herein. Five other genes were excluded due to lack of expression or poor relationships. Furthermore, we found that *IGF2BP1* was differentially expressed between original and selected chicken lines. But regrettably, there was insufficient RNA-sequencing data to compare the transcriptomic difference between individuals with different genotypes, the existing samples are heterozygous for haplotype or SNPs. In a word, the results of this study indicated that *IGF2BP1* or its upstream regulatory element was directionally selected during the IMF enhancement, with confirmation by transcriptional activity differences between the two chicken lines. 

## 5. Conclusions

Collectively, intensive selection for IMF in chickens has produced a pattern of genomic variant, reflected in lipid- and muscle-related pathways and genes that differed from unselected chickens. *IGF2BP1* was confirmed as a major gene affecting BrW in JX chickens. It’s worth noting that a refined haplotype and two conserved SNPs located in the up-stream of *IGF2BP1* have a strong effect on BrW, and the frequency change of which was consistent with the selection process. These results suggest novel targets for investigation of the genetic mechanisms that impact breast muscle development. Such an investigation will serve as the foundation upon which to improve chicken BrW.

## Figures and Tables

**Table 1:** Candidate genes in the significant region of chromosome 3 and 27.

Chromosome	Start	End	nSNP	Lead SNP	Alleles	MAF ^1^	β ^2^	Candidate Genes
27	6,086,729	6,339,862	62	chr27_6115361	A/G	0.48	−7.13	*IGF2BP1*, *GIP*, *SNF8*, *UBE2Z*, *ATPSMC1*, *CALCOCO2*, *HOXB1*, *HOXB2*, *HOXB3*, *HOXB4*, *HOXB5*, *HOXB6*, *HOXB7*, *HOXB8*, *HOXB9*, *HOXB13*, *SKAP1*, *gga-mir-196*, *gga-mir-10a*
3	72,191,174	72,191,174	1	chr3_72191174	A/G	0.14	9.19	*ENSGALG00000034564*

^1^ MAF indicates minor allele frequency. ^2^ Allele substitution effect was estimated by GEMMA.

## Data Availability

The genomic data could be accessed with CRA002643 and CRA002650, and the transcriptome data could be accessed with CRA001908 and CRA001334.

## Supplementary Materials

The following are available online at https://www.mdpi.com/article/10.3390/genes13010003/s1, Table S1: Phenotypic records of JX chickens. Table S2: Frequency difference of missense SNPs between original and selected population. Table S3: Annotation result of missense SNPs with significant frequency difference. Table S4: Pathways enrichment analysis based on genes with frequency difference missense SNPs. Table S5: Statistics for conservation score and frequency difference of high-impact SNPs. Table S6: Annotation result of high-impact SNPs. Table S7: Pathways enrichment analysis based on genes with high-impact SNPs. Table S8: Significant SNPs in refined region identified by GWAS for BrW. Table S9: The frequency of haplotypes formed by 31 SNPs. Table S10: DEGs identified in original and selected population. Figure S1: Pathway enrichment analysis for genes with missense SNPs of frequency difference. Figure S2: Correlation analysis between HOXB genes and BrW. Figure S3: Differentially expressed analysis for HOXB genes. Figure S4: Pattern of breast muscle development from embryo to post-hatching stages. Figure S5: Volcano plot of DEGs between original and selected population. Figure S6: Pathways enrichment analysis based on DEGs. Figure S7: GO terms related to muscle development.

## Abbreviations

BrW	Breast Muscle Weight
DEG	Differentially Expressed Gene
FC	Fold Change
GLM	General Linear Model
GWAS	Genome-wide Association Study
IMF	Intramuscular Fat
JX	Jingxing yellow chicken
LMM	Linear Mixed Model
SNP	Single Nucleotide Polymorphism
