# Supplementary figures and images for: Large-Scale Whole Genome Sequencing Study Reveals Genetic Architecture and Key Variants for Breast Muscle Weight in Native Chickens
Source: Genes (Basel). 2021 Dec 21;13(1):3. doi: 10.3390/genes13010003 (PMC8774586; doi:10.3390/genes13010003)

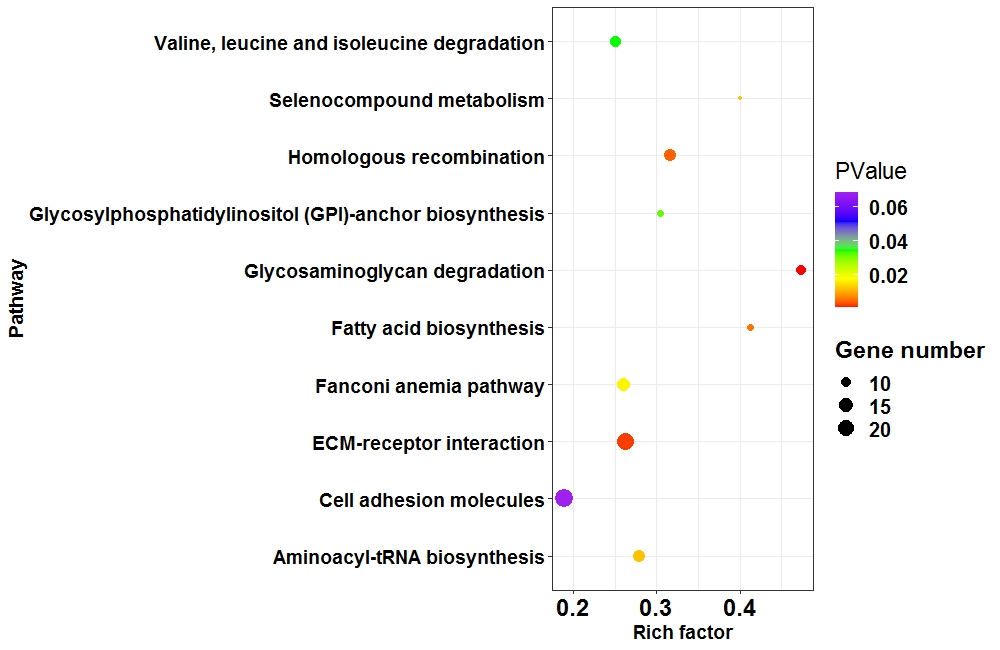

Supplement: Supplementary file 1 [file genes-13-00003-s001.zip › Supplement Figure S1.jpeg]

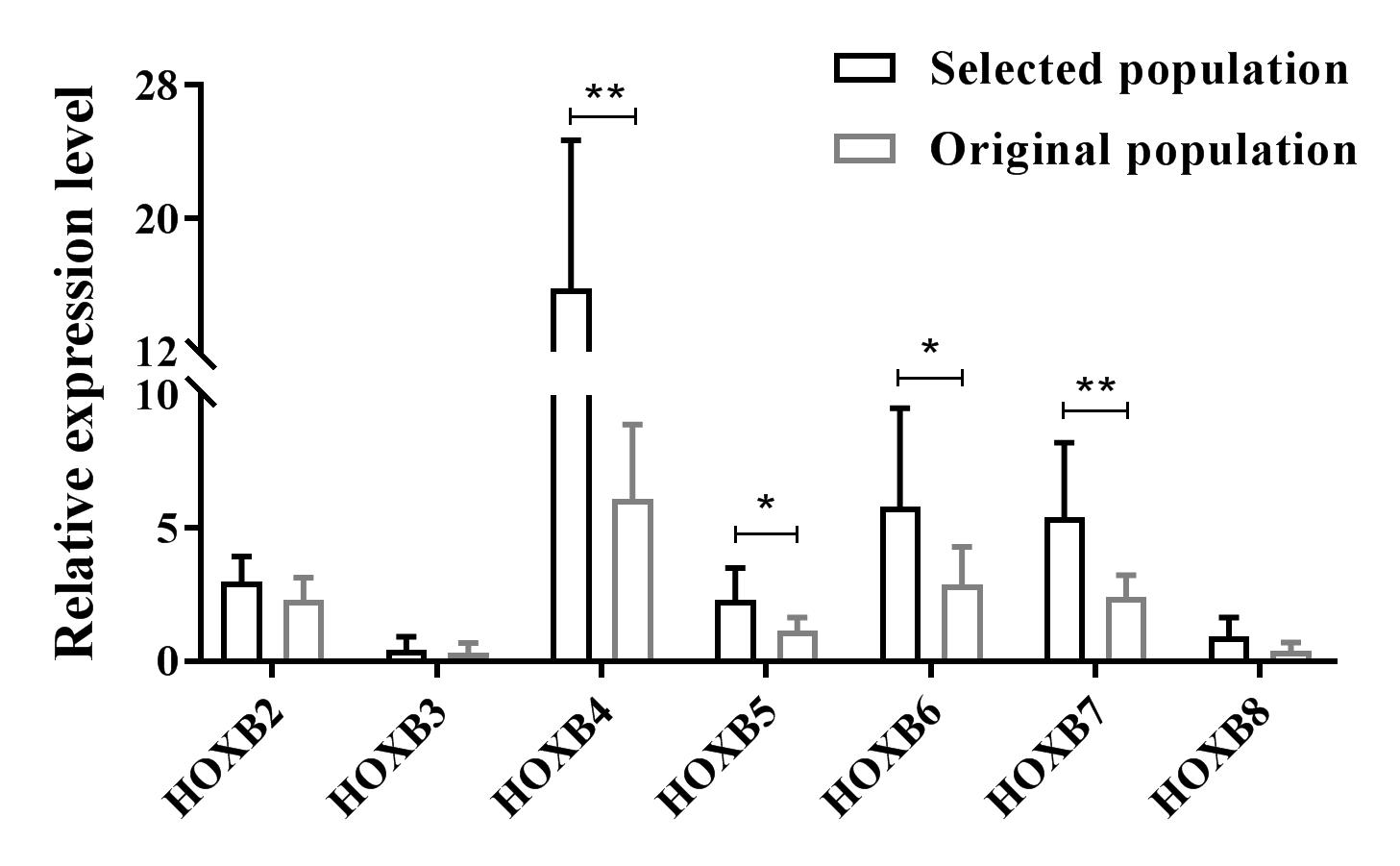

Supplement: Supplementary file 1 [file genes-13-00003-s001.zip › Supplement Figure S3.jpg]

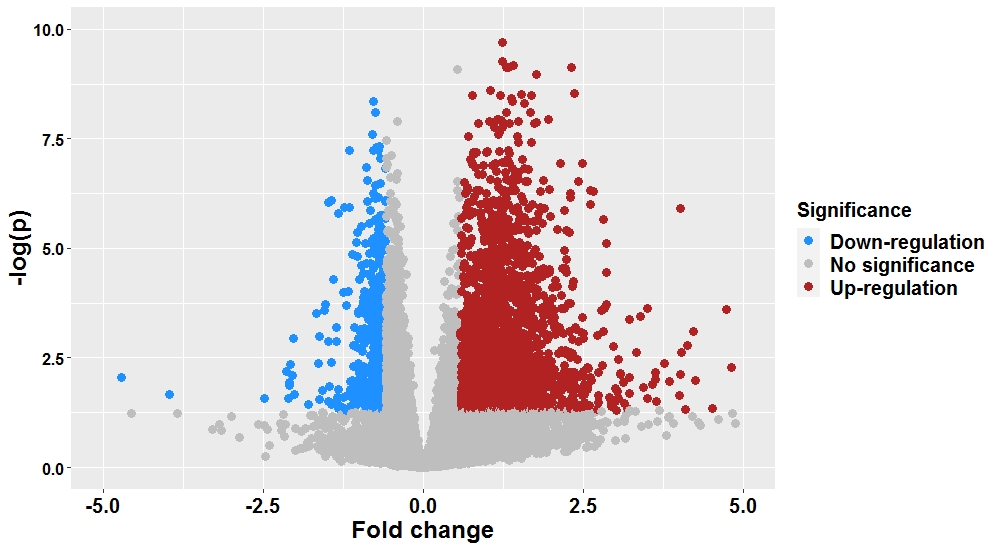

Supplement: Supplementary file 1 [file genes-13-00003-s001.zip › supplement Figure S5.jpeg]

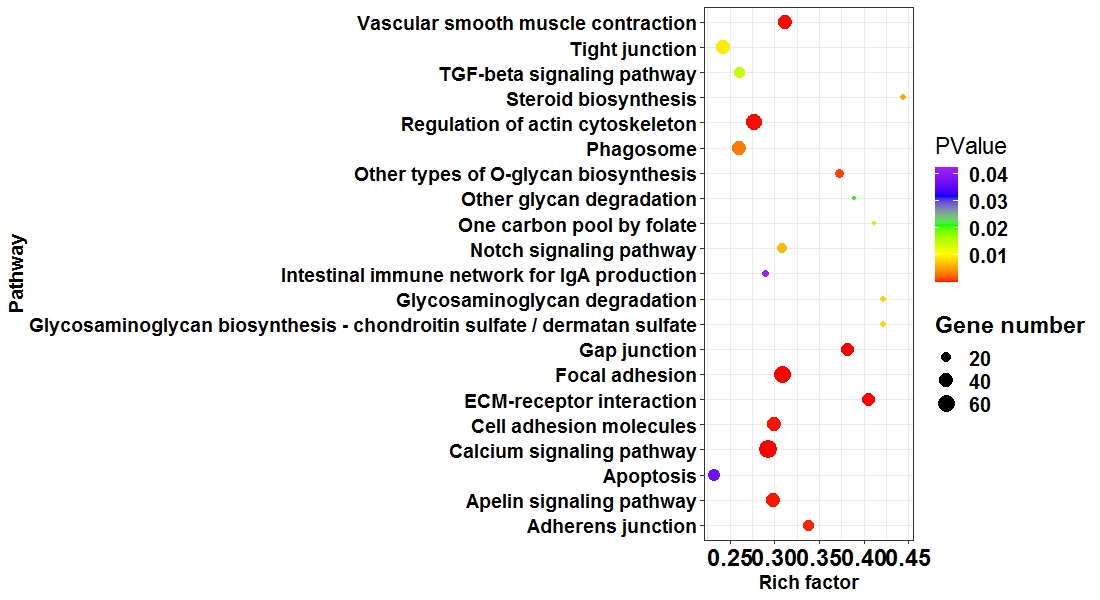

Supplement: Supplementary file 1 [file genes-13-00003-s001.zip › supplement Figure S6.jpeg]

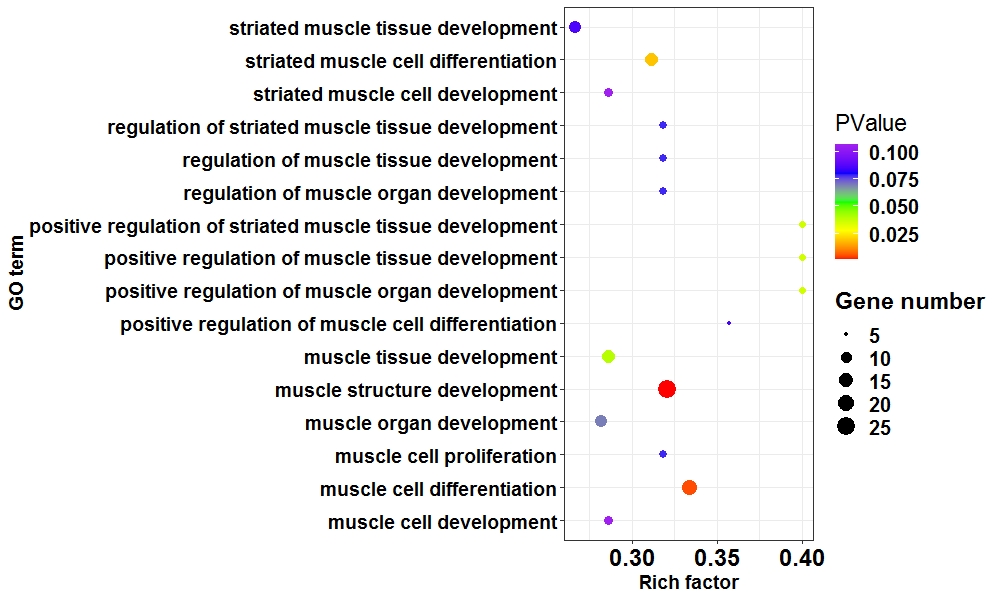

Supplement: Supplementary file 1 [file genes-13-00003-s001.zip › Supplement Figure S7.jpeg]
